# Electroacupuncture Alleviates Mechanical Allodynia of a Rat Model of CRPS-I and Modulates Gene Expression Profiles in Dorsal Root Ganglia

**DOI:** 10.3389/fneur.2020.580997

**Published:** 2020-10-30

**Authors:** Jie Wang, Xiaoli Zheng, Boyu Liu, Chengyu Yin, Ruixiang Chen, Xiaojie Li, Yuanyuan Li, Huimin Nie, Danyi Zeng, Xiaofen He, Yongliang Jiang, Jianqiao Fang, Boyi Liu

**Affiliations:** Key Laboratory of Acupuncture and Neurology of Zhejiang Province, Department of Neurobiology and Acupuncture Research, The Third Clinical Medical College, Zhejiang Chinese Medical University, Hangzhou, China

**Keywords:** RNA-sequencing, acupuncture, pain, immune response, CRPS-I

## Abstract

Complex regional pain syndrome type-I (CRPS-I) is chronic neurological disorder accompanied with devastating pain. Most conventional medical treatments lack effectiveness, making CRPS-I a challenging clinical condition. Electroacupuncture (EA) showed effectiveness in alleviating the pain symptoms of CRPS-I patients. However, the molecular mechanisms underlying EA's therapeutic effect are still not well-understood. Here, we established the rat chronic post-ischemic pain (CPIP) model to mimic CRPS-I and performed repetitive EA on bilateral hind limbs of the CPIP model rats. We then performed RNA-sequencing (RNA-Seq) to study the differences in gene expression, gene networks, and molecular pathways in ipsilateral DRGs innervating the hind limb of the CPIP model rats with and without repetitive EA treatment. Our results found that repetitive EA treatment significantly alleviated mechanical allodynia in bilateral hind limbs of CPIP model rats. RNA-Seq analysis indicated that EA modulated the expression of multiple genes and gene networks in the DRGs of CPIP model rats. Further bioinformatics analysis identified the up-regulation of an array of genes involved in biological process such as neutrophil chemotaxis and immune response in the DRGs of CPIP model rats after EA treatment. Thus, these results suggest that EA may alleviate pain response in CPIP model rats via regulating multiple genes. Our work may help to further advance the understandings of the molecular mechanisms underlying EA's therapeutic effects on CRPS-I and help to identify novel targets for CRPS-I treatment.

## Introduction

Complex regional pain syndrome (CRPS) is a chronic neurological disorder with clinical characters of spontaneous pain, hyperalgesia, limb edema, vasomotor instability, and impairment of motor function (1–3). It is subdivided into two categories: CRPS-I (without identifiable nerve injury) and CRPS-II (with identifiable nerve injury) (4, 5). CRPS-I is more prevalent than CRPS-II. Bone fractures, limb trauma, surgery, or ischemia are common triggers for CRPS-I (6, 7). Of note, pain is among the clinical features of CRPS-I that mostly affect the patients, which severely impairs life qualities. It is estimated that the prevalence of CRPS-I is around 30–40% among patients with stroke or bone fracture (8, 9). Unfortunately, there is still no effective medical treatment available for CRPS-I at present (10). Clinical trials have also failed to support the efficacy of many commonly used interventions, making it a challenging neurological disorder in clinic (7, 10). Therefore, alternative therapeutic options for CRPS-I pain are urgently needed.

In order to study the pain mechanisms of CRPS-I, the rat chronic post-ischemic pain (CPIP) model was developed by applying prolonged ischemia and reperfusion to the hind limb (11). The CPIP rat model developed several important characters that recapitulate the clinic features of CRPS-I, including early swelling and hyperemia in the hind limb, followed with chronic mechanical, chemical, and thermal pain hypersensitivities (11, 12). The CPIP rat model has been widely utilized for mechanistic studies of CRPS-I. Recently, we and other peers have identified several important mechanisms that are involved in CPIP-related pain, including peripheral TRPV1/TRPA1 channels, NMDA receptor, reactive oxygen species, spinal CSF1, CXCL12, and NLRP3 inflammasome, etc. (13–18).

One potential therapeutic option for CRPS pain is electroacupuncture (EA), which incorporates traditional manual acupuncture with modern electrotherapy. EA has shown efficacy for treating a variety of pain conditions (19). Recent study demonstrates that EA can significantly reduce pain symptoms and improve life quality of CRPS-I patient, suggesting EA may serve as an alternative method for relieving CRPS-I-related pain (20). Furthermore, our recent work screened the optimal EA frequency and identified 100 Hz as an effective and reliable method for alleviating the pain response of CPIP model rats (15). Nevertheless, the molecular mechanisms underlying EA's therapeutic effects on CRPS pain is still largely unknown.

Dorsal root ganglia (DRGs) contain sensory neurons that receive and convey pain signals from the peripheral to the spinal cord dorsal horn (21). Thus, DRGs have important roles in primary pain signal generation, integration, transduction, and peripheral sensitization (21, 22). In this study, we performed RNA-Seq to study differences in gene expression, gene networks, and molecular pathways in ipsilateral L4-L6 DRGs innervating the hind limb of CPIP model rats with and without 100 Hz EA treatment. We found that EA can modulate the expression of multiple genes in DRGs of CPIP model rats. Interestingly, our data identified the up-regulation of an array of genes involved in neutrophil chemotaxis and immune response in CPIP model rats after EA treatment. Thus, our work may help to further advance the understandings of the molecular mechanisms underlying EA's therapeutic effect on CRPS-I.

## Materials and Methods

### Animals

Male Sprague-Dawley rats (8–10 weeks, 300–320 g) were purchased from Shanghai Laboratory Animal Center, Chinese Academy of Sciences. Rats were randomly allocated, and five rats were housed per cage. The rats were housed in the Laboratory Animal Center of Zhejiang Chinese Medical University accredited by the Association for Assessment and Accreditation of Laboratory Animal Care (AAALAC) under standard environmental conditions (12 h light–dark cycles and 24 ± 2°C). Food and water were provided *ad libitum*. The rats were given minimum of 1 week to adapt to the new environment before experiment. All animal care and experimental studies were approved by the Laboratory Animal Management and Welfare Ethical Review Committee of Zhejiang Chinese Medical University (Permission Number: ZSLL-2017-183).

### CPIP Rat Model Establishment

CPIP was established through prolonged hind paw ischemia and reperfusion as described previously (11, 12). Anesthesia was induced in all rats with an intraperitoneal injection of 50 mg/kg of sodium phenobarbital and was maintained with an infusion of sodium phenobarbital at 20 mg/kg/h. A Nitrile 70 Durometer O-ring with a 7/32″ (5.5 mm) internal diameter was slide into the larger side of a 1.5 mL Eppendorf tube (with the snap-cap cut off before use). The O-ring was cut off 3 h later for reperfusion. Sham rats received the same anesthetic procedure but the ankle was surrounded with a cut O-ring which did not block blood flow.

### EA Treatment

The procedure of EA was carried out according to our previous study (23). Briefly, rats were loosely immobilized, and acupuncture needles of 0.25 mm in diameter were inserted at a depth of 5 mm into bilateral Zusanli (ST36, 5 mm lateral to the anterior tubercule of the tibia) and Kunlun (BL60, at the ankle joint level and between the tip of the external malleolus and calcaneus) acupoints. The needles were connected with HANS acupuncture point nerve stimulator (HANS-200A Huawei Co., Ltd., Beijing, China). The parameters were set as follows: 100 Hz, square wave current output (pulse width: 0.2 ms). Intensities ranging from 0.5 to 1.5 mA (increased by 0.5 mA every 10 min, for a total of 30 min) were delivered for a period of 30 min. The treatment was conducted once daily for 10 consecutive days.

### Determination of Mechanical Allodynia

Before baseline test, rats were habituated to the test environment daily for a consecutive 3 days. Rats were individually placed in transparent Plexiglas chambers on an elevated mesh floor and were habituated for 30 min before the test. The mechanical allodynia was determined using a series of von Frey filaments (UGO Basile, Italy) applied perpendicularly to the mid-plantar surface of the hind paws, with sufficient force to bend the filament slightly for 3–5 s according to methods previously used (14, 24). An abrupt withdrawal of the paw and licking and vigorously shaking in response to stimulation were considered pain-like responses. The threshold was determined using the up-down testing paradigm, and the 50% paw withdrawal threshold (PWT) was calculated by the non-parametric Dixon test (25). The behavior tests are conducted by an experimenter blinded to experimental conditions.

### Determination of Hind Paw Swelling

Swelling was observed as an increase in hind paw diameter, as measured by a digital caliper, and was calculated as the difference between the basal value and the test value as in our previous study (13). Each rat was measured 3 times, and the mean value was calculated.

### Tissue Collection and RNA Extraction

At day 10, rats were deeply anesthetized with sodium pentobarbital (40 mg/kg) and were perfused transcardially with 200 mL 0.9% saline (4°C). After the perfusion, the right (ipsilateral) side L4-6 DRG segments were collected and were immediately preserved in the RNA later solution (Invitrogen, Carlsbad, USA). Total RNA was extracted using Trizol reagent (Invitrogen, Carlsbad, USA) following the manufacturer's instructions and treated with DNase I to degrade contaminating DNA. The purity and concentration of the samples was assessed by the Nanodrop Spectrophotometer (NanoDrop Products, CA, USA), and the RNA integrity was assessed by the Agilent 2100 Bioanalyzer (Agilent Technologies, Palo Alto, CA).

### RNA-Seq Library Establishment and RNA-Seq

Total mRNAs from three rats of each group were isolated and used to construct sequencing libraries. DNase I was used to degrade double-stranded and single-stranded DNA contaminant in RNA samples. mRNA molecules were purified from total RNA using oligo(dT)-attached magnetic beads. mRNA were fragmented into small pieces using fragmentation reagent. First-strand cDNA was generated using random hexamer-primed reverse transcription, followed by a second-strand cDNA synthesis. The synthesized cDNA was subjected to end-repair and then was 3′ adenylated. Adaptors were ligated to the ends of these 3′ adenylated cDNA fragments. This process is to amplify the cDNA fragments with adaptors from previous step. PCR products are purified with the SPRI beads, and dissolved in EB solution. The double stranded PCR products were heat denatured and circularized by the splint oligo sequence. The single-strand circle DNA (ssCir DNA) was formatted as the final library. Library was validating on the Agilent Technologies 2100 bioanalyzer. The library was amplified with phi29 to make DNA nanoball (DNB) which have more than 300 copies of one molecular. The DNBs were loaded into the patterned nanoarray and single end 50 base reads were generated in the way of sequenced by synthesis. Finally, the fragments were enriched by PCR amplification to construct a library ready for sequencing using BGISEQ-500 by BGI Group (Shenzhen, China).

### Bioinformatics Analysis

Primary sequencing data produced by RNA-Seq (raw reads) were subjected to quality control (QC). The information of total reads and mapping ratio reads were shown in Table 1. Raw reads were filtered into clean reads by internal software SOAPnuke (version 1.5.2), followed by: Remove reads in which unknown bases (N) are more than 10%; Remove reads with adaptors; Remove low quality reads (we define the low quality read as the percentage of base which quality is <15 and >50% in a read). QC of alignment was performed to determine if re-sequencing was needed. If the alignment result passed QC, downstream analysis including gene expression, differentially expressed genes, cluster analysis, Gene Ontology (GO) enrichment analysis, Kyoto Encyclopedia of Genes and Genomes (KEGG) pathway enrichment analysis, etc. was proceeded with as we described previously (26).

**Table 1:** The information of total reads and mapping ratio for Sham, CPIP, and CPIP+EA groups in RNA-Seq.

**Sample**	**Total raw reads**	**Total clean reads**	**Clean reads Q30 (%)**	**Clean reads ratio (%)**	**Total mapping ratio (%)**
	**(Mb)**	**(Mb)**			
Sham1	21.94	21.82	89.93	99.44	95.36
Sham2	21.94	21.8	90.23	99.35	95.39
Sham3	21.94	21.78	90.77	99.25	95.48
CPIP1	21.94	21.82	90.21	99.45	95.52
CPIP2	21.94	21.83	89.89	99.48	95.53
CPIP3	21.94	21.82	89.46	99.45	94.95
CPIP+EA1	21.94	21.87	91.9	99.68	95.35
CPIP+EA2	21.94	21.87	92.19	99.68	95.05
CPIP+EA3	21.94	21.81	92.69	99.41	94.97

### Cluster Analysis and Screening of Differentially Expressed Genes

Distances of expressed genes were calculated using the Euclidean method (27). The sum of the squared deviations algorithm was used to calculate distance. The cluster analysis and heat map visualization of gene expression patterns was performed using the “pheatmap” package in the R software of Bioconductor. Differentially expressed mRNAs with statistical significance were identified through Volcano Plot filtering. The threshold required for the results to be considered significant was as follows: *Q* < 0.01 and fold change ≥1.5 or ≤0.667.

### Functional Enrichment Analysis of DEGs

Functional enrichment analysis was performed by functional annotation package “clusterProfiler” in R studio software (RStudio, Boston, MA, USA). GO and KEGG enrichment analysis was also conducted. For each enriched function term, *P*-value of enriched functions and the *P*-value by multiple testing corrections were calculated by “clusterProfiler” package in R studio software. The GO functional and KEGG pathway enrichment analysis were performed for DEGs using the Database for Annotation, Visualization and Integrated Discovery (DAVID) online tools (http://www.geneontology.org and http://www.genome.jp/kegg).

### Real-Time Quantitative PCR (qPCR)

The extracted total RNA from the DRGs was reverse transcribed into cDNA using random hexamers primers (TaKaRa Bio Inc., Shiga, Japan) according to the manufacturer's instruction. The sequences of all primers used were shown in Table 2. qPCR was performed using the Fast Start Universal SYBR Green Master kit (TaKaRa Bio Inc., China) 20 μL reaction system according to the manufacturer's protocol by LightCycler480 System (Roche, America). Each reaction was performed in triplicates and normalized to β-actin gene expression. The CT value of each well was determined using the LightCycler480 System software and the average of the triplicates was calculated. The relative quantification was determined by the ΔΔCT method (28, 29).

**Table 2:** Sequences of the primers used for qPCR validation of RNA-Seq data.

**Gene name**	**Gene ID**	**Primer sequence (5′-3x02032;)**	**Amplicon size (bp)**
*Pf4*	360918	F: 5′-CTGCTTCTTCTGGGTCTGCTGTTG-3′	140
		R: 5′-AGGCTGGTGATGCGTTTGAGATG-3′	
*RT1-A1*	24973	F: 5′-CCTCCTCCATCAACCGATTCCAAC-3′	107
		R: 5′-ACAACAGTCACCACAGCTCCAATG-3′	
*Pglyrp1*	84387	F: 5′-CTCCGTGCTGCCCTAAATCTTCTG-3′	127
		R: 5′-TGATCTCGTAGAGCTGGTCACCTG-3′	
*Ccl9*	360579	F: 5′-AGCCCTTTCATACTGCCCTCTCC-3′	117
		R: 5′-GCTGATGTCCTTGCTCTGCCTTC-3′	
*Il1rn*	60582	F: 5′-TACCTTCATCCGCTCCGAGACAG-3′	128
		R: 5′-AGGGCTCTTTTGGTGTGTTGGTG-3′	
*Ccl3*	25542	F: 5′-AGAGGCAGCGAGTACCAGTCC-3′	184
		R: 5′-CATAGGAGAAGCAGCAGGCAGTC-3′	

### Protein-Protein Interaction (PPI) Network Analysis

The Search Tool for the Retrieval of Interacting Genes (STRING) is used to provide information regarding predicted and experimental interactions of proteins and the prediction method of this database is from neighborhood, gene fusion, co-occurrence, co-expression experiments, databases, and text mining. By setting the Combination score >0.7 as the reliability threshold value, the web based STRING database (http://string-db.org/) was used to produce PPI predictions after uploading the union gene list to the search bar (30). Based on the interplayed relationships, a PPI network was established and then visualized using the Cytoscape software (31). The connectivity degree of each protein, namely the number of proteins it connected, was calculated to evaluate its importance in this network.

### Statistical Analysis

One- or two-way ANOVA followed by Tukey's *post-hoc* test was used for comparison among groups ≥3. Student's *t*-test was used for comparisons between two groups. Data in graphs are expressed as means ± SEM. Comparison is considered significantly different if the *p* < 0.05.

## Results

### The Establishment of the CPIP Rat Model and Nocifensive Behavior Evaluation

**Figure 1:**
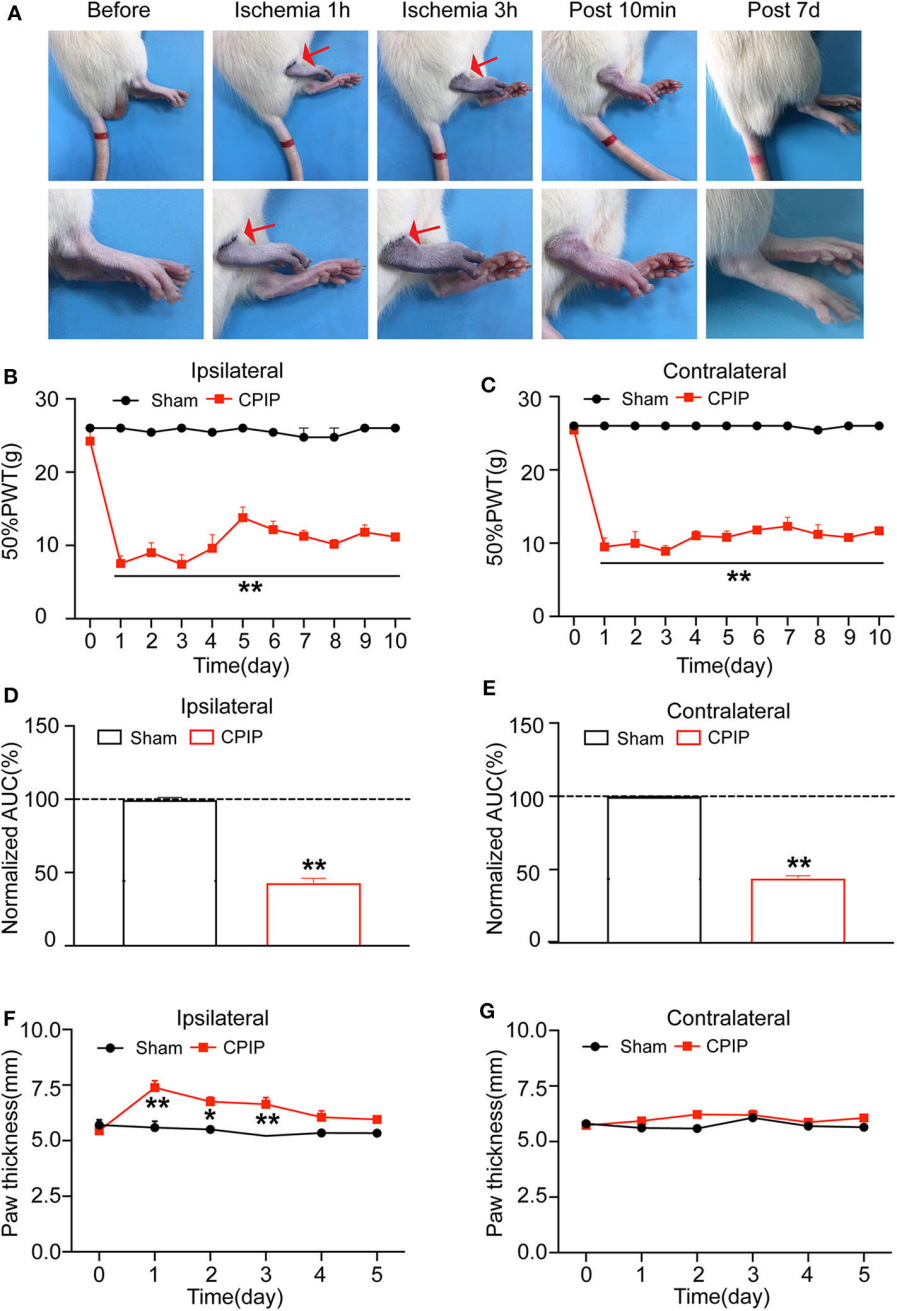
The rat chronic post-ischemic pain model (CPIP) exhibited long-lasting bilateral mechanical allodynia. **(A)** Representative photographs of rat hind paw taken during CPIP model establishment, 10 min and 7 day after model establishment. The red arrow indicates the paw with the O-ring (ipsilateral side). **(B)** 50% paw withdraw threshold (PWT) of ipsilateral hind paw. **(C)** 50% PWT of contralateral hind paw. **(D)** Summary of the normalized area under the curve (AUC) as in **(B)**. **(E)** Summary of the normalized AUC as in **(C)**. **(F)** Thickness evaluation of the ipsilateral hind paw. **(G)** Thickness evaluation of the contralateral hind paw. *n* = 6 rats/group. **p < 0.01 vs. Sham group. Student's *t*-test or two-way ANOVA followed by Tukey *post-hoc* test was used for statistical analysis. *p < 0.05.

At beginning, we set to establish the rat CPIP model, which mimics human CRPS-I, according to methods described before (11, 12). The hind paw of the CPIP model rats exhibited cyanosis, a sign of tissue hypoxia, and edema when the O-ring was placed (Figure 1). After reperfusion, the hind paw was filled with blood. The edema persisted 3 days and gradually returned back to normal. Furthermore, the CPIP model rats developed obvious mechanical allodynia in both ipsilateral and contralateral hind paws, which persisted until 10 days of our observation time frame. The above observations are consistent with previous results and indicated successful establishment of the CPIP rat model (12).

### EA Significantly Ameliorated Mechanical Allodynia of CPIP Model Rats

**Figure 2:**
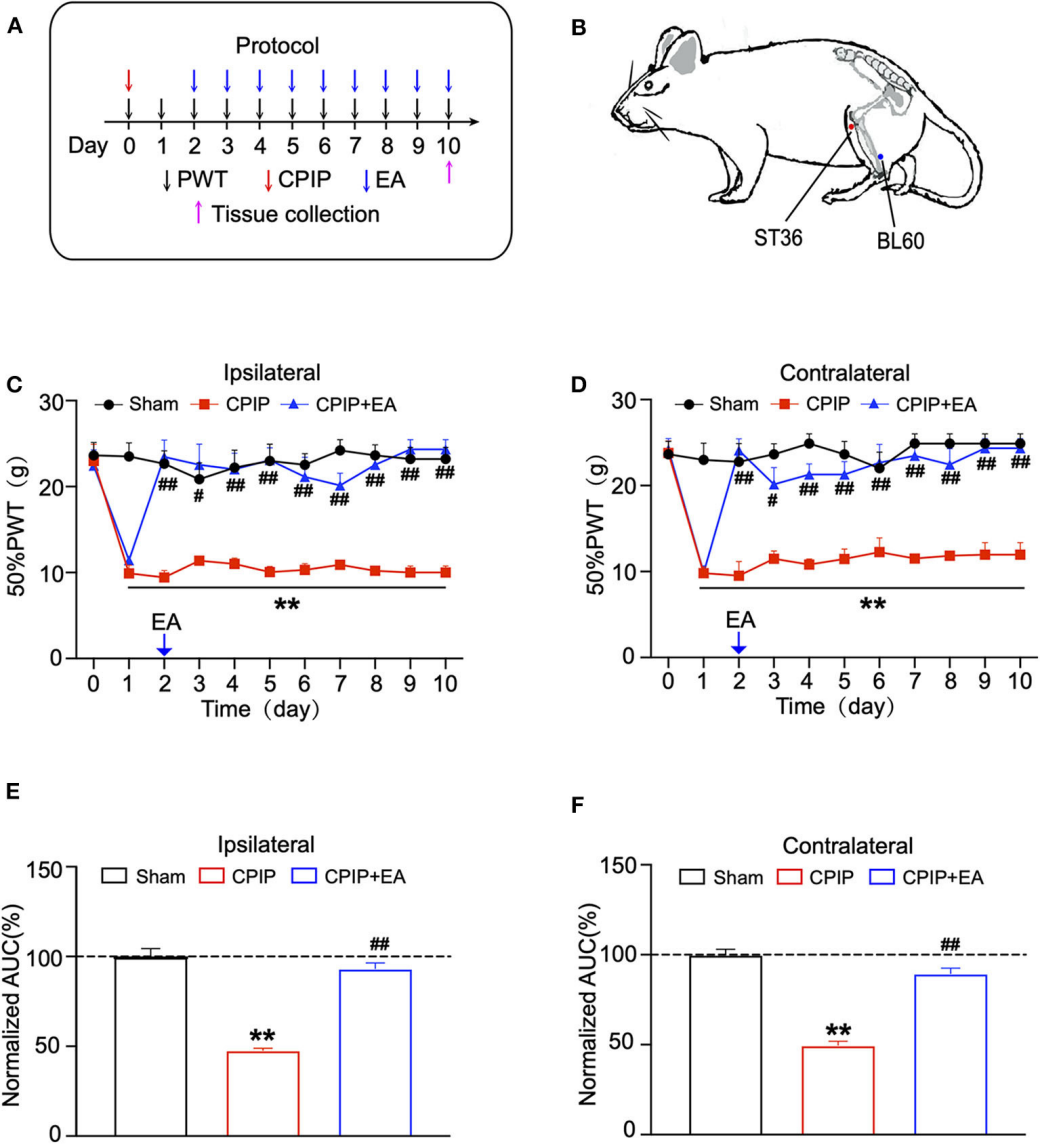
Evaluation of the anti-allodynic effects of electroacupuncture (EA) on CPIP model rats. **(A)** Experimental protocol for EA treatment. **(B)** Schematic picture of the locations of acupoints ST36 and BL60 on rat. **(C)** The effect of bilateral 100 Hz EA on 50% PWT of ipsilateral hind paws of CPIP rats. **(D)** The effect of bilateral 100 Hz EA on 50% PWT of contralateral hind paws of CPIP rats. **(E)** Summary of the normalized AUC as in **(C)**. **(F)** Summary of the normalized AUC as in **(D)**. *n* = 6 rats/group. **p < 0.01 vs. Sham group. ^#^*p* < 0.05 and ^##^*p* < 0.01 vs. CPIP group. One-way ANOVA or two-way ANOVA followed by Tukey *post-hoc* test was used for statistical analysis.

We proceeded to examine the effects of EA on the mechanical allodynia of CPIP model rats. In our recent study, we screened the optimal EA frequency for CPIP treatment and found that daily 100 Hz EA treatment showed effective and reliable analgesic effect on mechanical allodynia of CPIP model rats (15). Therefore, in the present study, we selected 100 Hz EA for the treatment. The procedure of the EA treatment was indicated in Figures 2A,B. EA significantly ameliorated mechanical allodynia in both ipsilateral and contralateral hind paws of CPIP model rats on each time point for 10 days of our observation time frame (Figures 2C,D). The analysis of the area under the curve (AUC) further indicated an overall anti-allodynic effect on bilateral hind paws (Figures 2E,F). These data confirmed that 100 Hz EA is able to attenuate the mechanical allodynia of CPIP model rats.

### Exploring the Differentially Expressed Genes in Ipsilateral Dorsal Root Ganglions (DRGs) After EA Treatment

**Figure 3:**
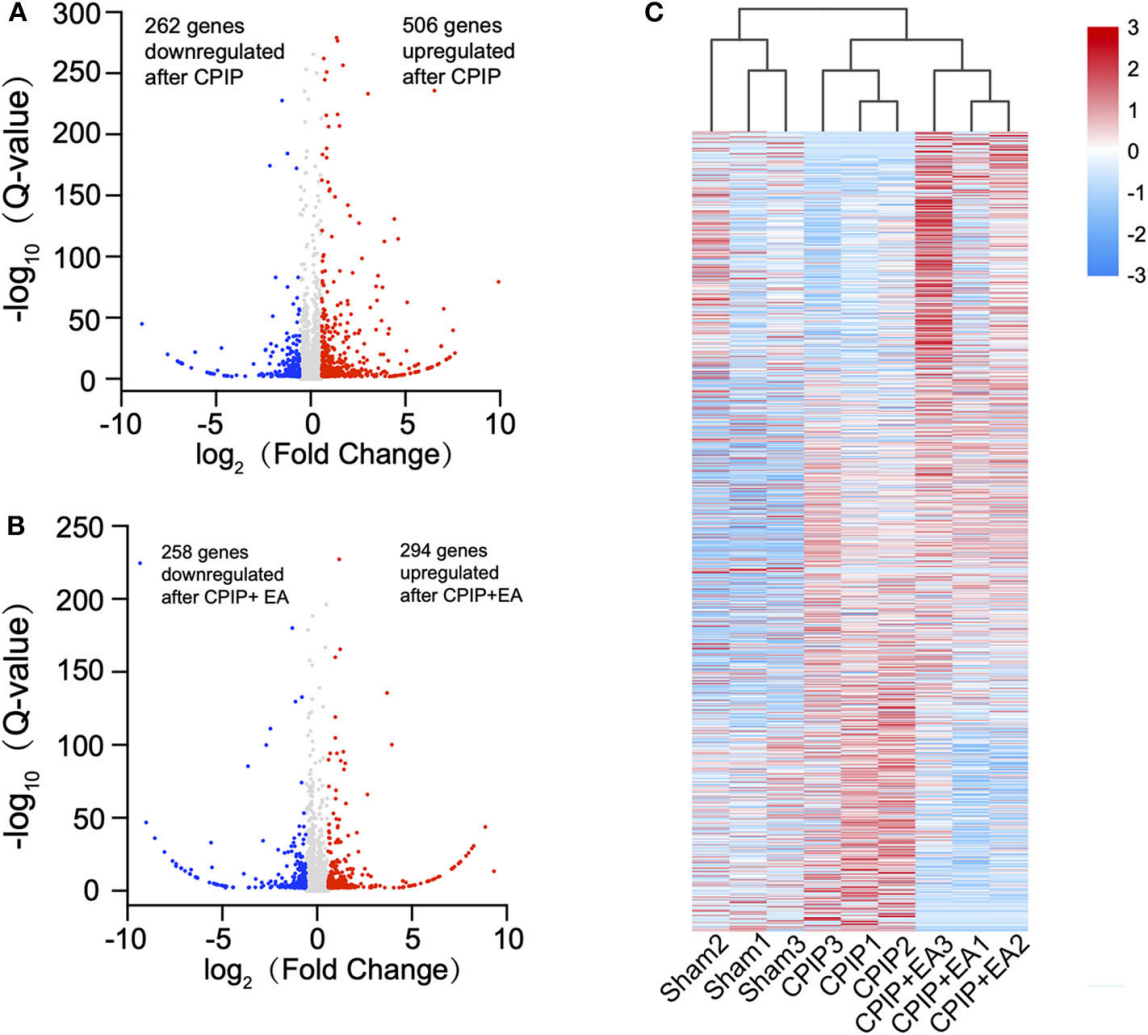
RNA-sequencing (RNA-Seq) reveals transcriptome profiles of gene expression changes in the dorsal root ganglion (DRGs) induced by CPIP and EA treatment. **(A)** Volcano plot showing gene expression profiles in DRGs of CPIP group vs. Sham group. **(B)** Volcano plot showing gene expression profiles in DRGs of CPIP+EA group vs. CPIP group. Red and blue spots indicate up and down regulated DEGs, respectively, whereas gray spots indicate non-DEGs. **(C)** Heat map illustration of the hierarchical clustering of DEGs from Sham, CPIP, and CPIP+EA groups.

To determine the effects of EA on gene expression in DRGs of the ipsilateral side of CPIP model rats, we performed RNA-Seq to explore gene expression profiles of ipsilateral L4–6 DRGs 10 days after CPIP model establishment. RNA-Seq generated approximately 21.9 million raw reads in each sample. The ratio of clean reads reached above 99.0% (Table 1). Over 90% of bases had a quality score ≥Q30 and >95% of the clean reads were mapped to rat genome. In all, 20,413 genes were successfully mapped and identified from RNA-Seq. The differentially expressed genes (DEGs) of CPIP group vs. sham group and CPIP+EA group vs. CPIP group are displayed in the volcano plot as in Figures 3A,B. In all, 768 DEGs, including 506 up- and 262 down-regulated, were identified in CPIP vs. sham group, whereas 552 DEGs, including 294 up- and 258 down-regulated, were identified in CPIP+EA vs. CPIP group (Figures 3A,B). The top 20 up- and down-regulated DEGs identified in CPIP+EA vs. CPIP group were further illustrated in Tables 3, 4.

**Table 3:** The detailed information of the top 20 upregulated DEGs.

**Gene ID**	**Gene symbol**	**Full gene name**	**Log_**2**_ fold change**	***Q*-value**
			**(CPIP+EA/CPIP)**	
114021	Ebna1bp2	EBNA1 binding protein 2	9.33	3.37E-14
100912571	LOC100912571	Eukaryotic translation initiation factor 4 gamma 1-like	8.87	1.47E-44
108349606	LOC108349606	60S ribosomal protein L7a-like	8.27	1.71E-31
103691556	LOC103691556	Mothers against decapentaplegic homolog 9	8.17	1.04E-29
103690057	LOC103690057	Low-density lipoprotein receptor class A domain-containing protein 3-like	7.99	1.05E-26
297695	Wbp11	WW domain binding protein 11	7.86	1.00E-24
289881	Dr1	Down-regulator of transcription 1	7.57	7.08E-21
103694874	LOC103694874	Stromelysin-3	7.52	3.31E-20
313450	Rnf113a1	Ring finger protein 113A1	7.46	1.57E-19
293668	Brms1	BRMS1, transcriptional repressor, and anoikis regulator	7.24	3.79E-17
498109	Polr2h	RNA polymerase II subunit H	7.19	1.43E-16
122772	Rps20	Ribosomal protein S20	7.14	3.63E-16
100911713	LOC100911713	Protein C10-like	7.10	1.00E-15
100912009	LOC100912009	Zinc finger protein 844-like	6.47	9.60E-11
100910497	LOC100910497	Paired immunoglobulin-like type 2 receptor alpha-like	6.39	3.42E-10
300802	Aph1b	Aph-1 homolog B, gamma secretase subunit	6.22	3.34E-09
100911725	LOC100911725	6-phosphofructo-2-kinase/fructose-2,6-bisphosphatase 4-like	6.09	1.70E-08
108348131	Slc40a1	Solute carrier family 40 member 1	5.91	1.32E-07
100910678	LOC100910678	Coiled-coil domain-containing protein 72-like	5.84	2.88E-07
108348103	LOC108348103	Serine protease inhibitor Kazal-type 5-like	5.80	4.27E-07

*CPIP, chronic post-ischemia pain; EA, Electroacupuncture*.

**Table 4:** The detailed information of the top 20 downregulated DEGs.

**Gene ID**	**Gene symbol**	**Full gene name**	**Log_**2**_ fold change**	***Q*-value**
			**(CPIP+EA/CPIP)**	
316632	Ndufa10l1	NADH dehydrogenase (ubiquinone) 1 alpha subcomplex 10-like 1	−9.33	2.45E-225
364879	Isoc1	Isochorismatase domain containing 1	−9.00	1.03E-47
103693780	LOC103693780	2-oxoglutarate dehydrogenase, mitochondrial-like	−8.55	5.90E-37
100910581	LOC100910581	Protein phosphatase 1 regulatory subunit 35-like	−8.04	1.93E-27
108348109	Pdcd5	Programmed cell death 5	−7.61	2.69E-21
100359951	LOC100359951	Ribosomal protein S20-like	−7.43	3.96E-19
691543	Katnbl1	Katanin regulatory subunit B1 like 1	−7.43	1.29E-17
100912618	LOC100912618	Ubiquitin-conjugating enzyme E2 variant 1-like	−7.17	2.53E-16
293862	Fam50a	Family with sequence similarity 50, member A	−7.16	2.93E-16
100910616	Hmgn5	High mobility group nucleosome binding domain 5	−6.96	2.11E-14
100910177	LOC100910177	Dolichol-phosphate mannosyltransferase subunit 3-like	−6.71	2.55E-12
100909795	LOC100909795	Colorectal mutant cancer protein-like	−6.70	3.02E-12
292780	Alkbh6	alkB homolog 6	−6.64	2.99E-15
100361850	LOC100361850	Zinc finger protein 74 (Cos52)-like	−6.40	3.30E-10
108348082	LOC108348082	Dual specificity mitogen-activated protein kinase kinase 3-like	−6.39	3.85E-10
100910446	LOC100910446	Syntaxin-7-like	−6.37	4.76E-10
102550530	LOC102550530	MARCKS-related protein-like	−6.35	6.45E-10
287942	Crkl	CRK like proto-oncogene, adaptor protein	−6.32	1.03E-09
29257	Rpl9	ribosomal protein L9	−6.31	1.20E-09
304507	Oas1i	2′-5′ oligoadenylate synthetase 1I	−5.6777009	1.49E-06

*CPIP, chronic post-ischemia pain; EA, electroacupuncture*.

These DEGs were further summarized in the heat map and hierarchical clustering analysis was performed. We found that the samples from sham group, CPIP model group, and CPIP+EA group were clustered into separate groups, indicating a clear segregation between but not within these 3 groups (Figure 3C). This data indicated that there are significant gene expression changes in ipsilateral DRGs of CPIP model rats vs. sham rats, whereas EA treatment can modify gene expressions in CPIP model rats.

### The Analysis of DEGs in DRGs of CPIP Model Rats Receiving EA Treatment

**Figure 4:**
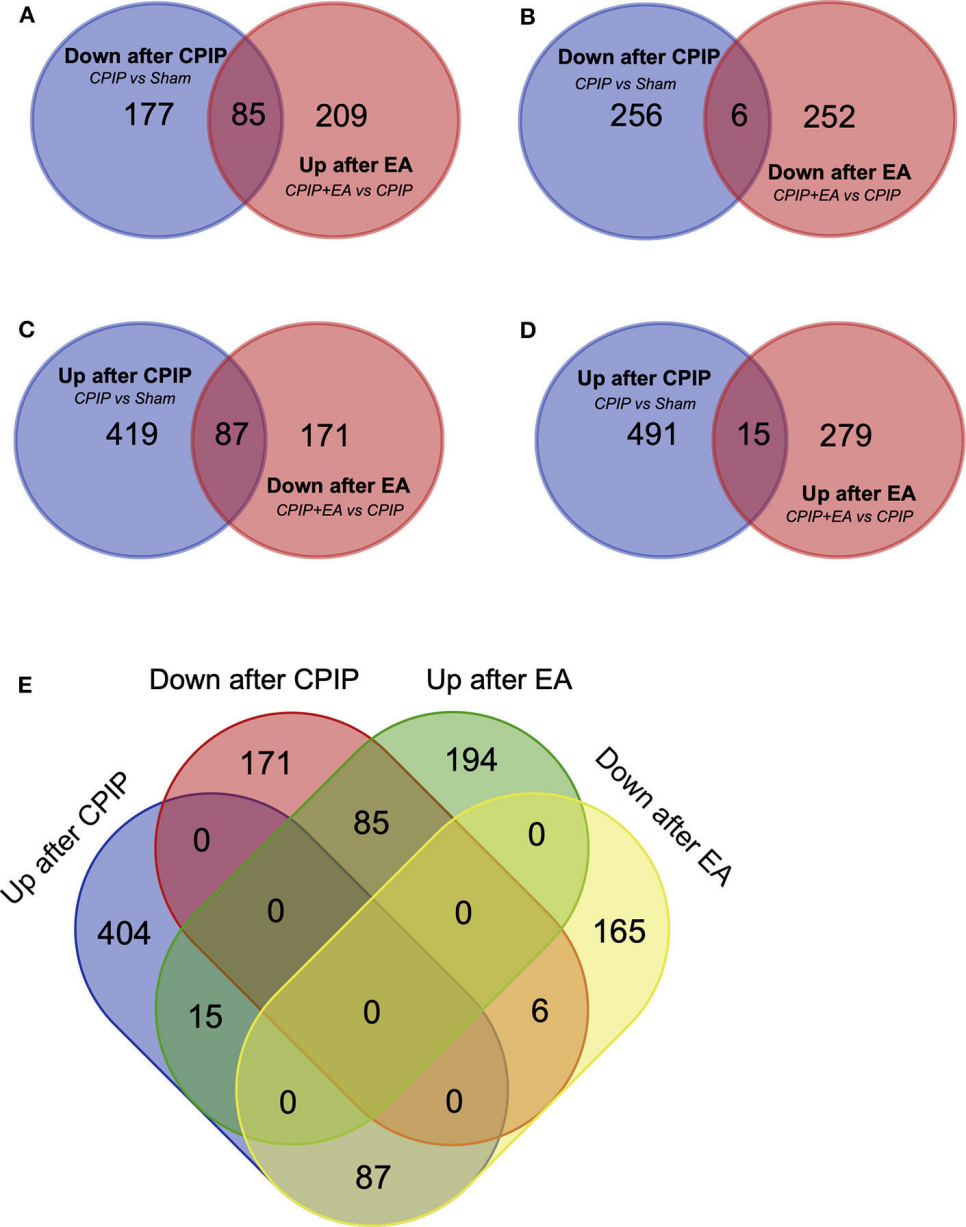
Overlapping of DEGs with genes involved in Sham, CPIP and CPIP+EA group. **(A–E)** Venn diagrams show overlaps of DEGs between different experimental groups. Eighty-five genes were decreased in CPIP group but increased in CPIP+EA group. Six genes were decreased in both CPIP and CPIP+EA group. Eighty-seven genes were increased in CPIP group but decreased in CPIP+EA group. Fifteen genes were increased in both CPIP and CPIP+EA group.

Venn analysis was performed to study the overlapping of DEGs among groups. Venn diagram showed that among the 262 DEGs that were down-regulated in CPIP group, 85 were reversed, whereas 6 were further down-regulated by EA treatment (Figures 4A,B, Supplementary Table 1). Among the 506 DEGs that were up-regulated in CPIP group, 87 were reversed, whereas 15 were further up-regulated by EA treatment (Figures 4C,D, Supplementary Table 2). Figure 4E further summarized the overall Venn diagram results showing the overlapping DEGs among groups.

**Figure 5:**
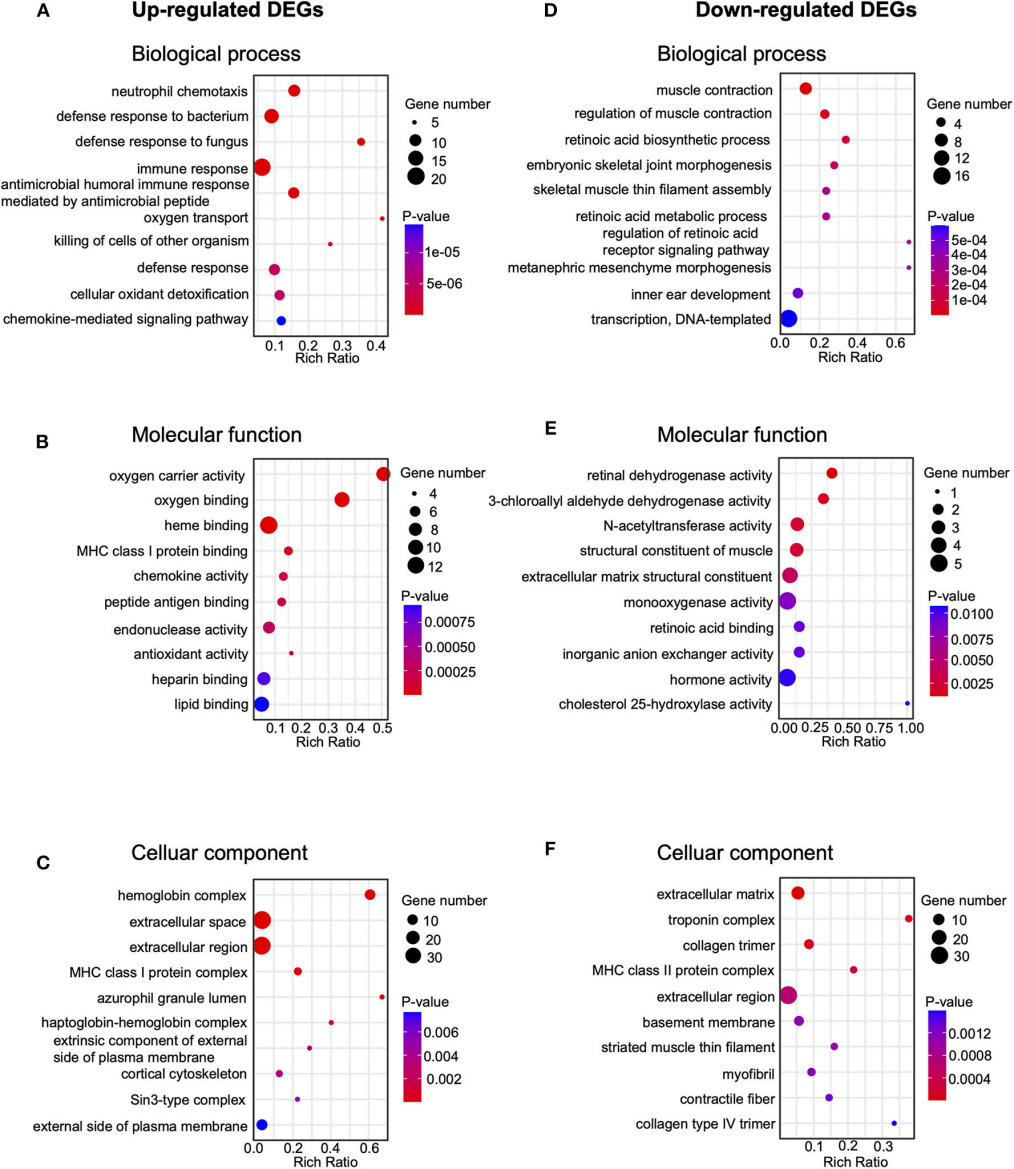
Gene ontology (Go) pathway analysis of DEGs. **(A–C)** Bubble plots showing the top 10 significant biological processes, molecular functions and cellular components of upregulated DEGs. **(D–F)** Bubble plots showing the top 10 significant biological processes, molecular functions, and cellular components of downregulated DEGs. Larger bubbles indicate higher number of genes. The color of each bubble reflects the significance (*p*-value).

In order to further explore the mechanisms underlying EA's therapeutic effects, we carried out GO analysis of the DEGs that are up- or down-regulated in CPIP+EA vs. CPIP group of rats. GO analysis showed that the most significantly enriched biological process of upregulated DEGs was neutrophil chemotaxis, followed by defense response to bacterium and fungus and immune response, etc. (Figure 5A, Supplementary Table 3). The most significantly enriched molecular function of upregulated DEGs included oxygen carrier activity, oxygen binding, and heme binding, etc. (Figure 5B, Supplementary Table 3). The most significantly enriched cellular function of upregulated DEGs included hemoglobin complex, extracellular space and extracellular region, etc. (Figure 5C, Supplementary Table 3). In contrast, the most significantly enriched biological process of down-regulated DEGs included muscle contraction, regulation of muscle contraction and retinoic acid biosynthetic process, etc. (Figure 5D, Supplementary Table 3). The most significantly enriched molecular function of downregulated DEGs included retinal dehydrogenase activity, 3-chloroallyl aldehyde dehydrogenase activity, and N-acetyltransferase activity, etc. (Figure 5E, Supplementary Table 3). The most significantly enriched cellular function of downregulated DEGs included extracellular matrix, troponin complex, and collagen trimer, etc. (Figure 5F, Supplementary Table 3).

**Figure 6:**
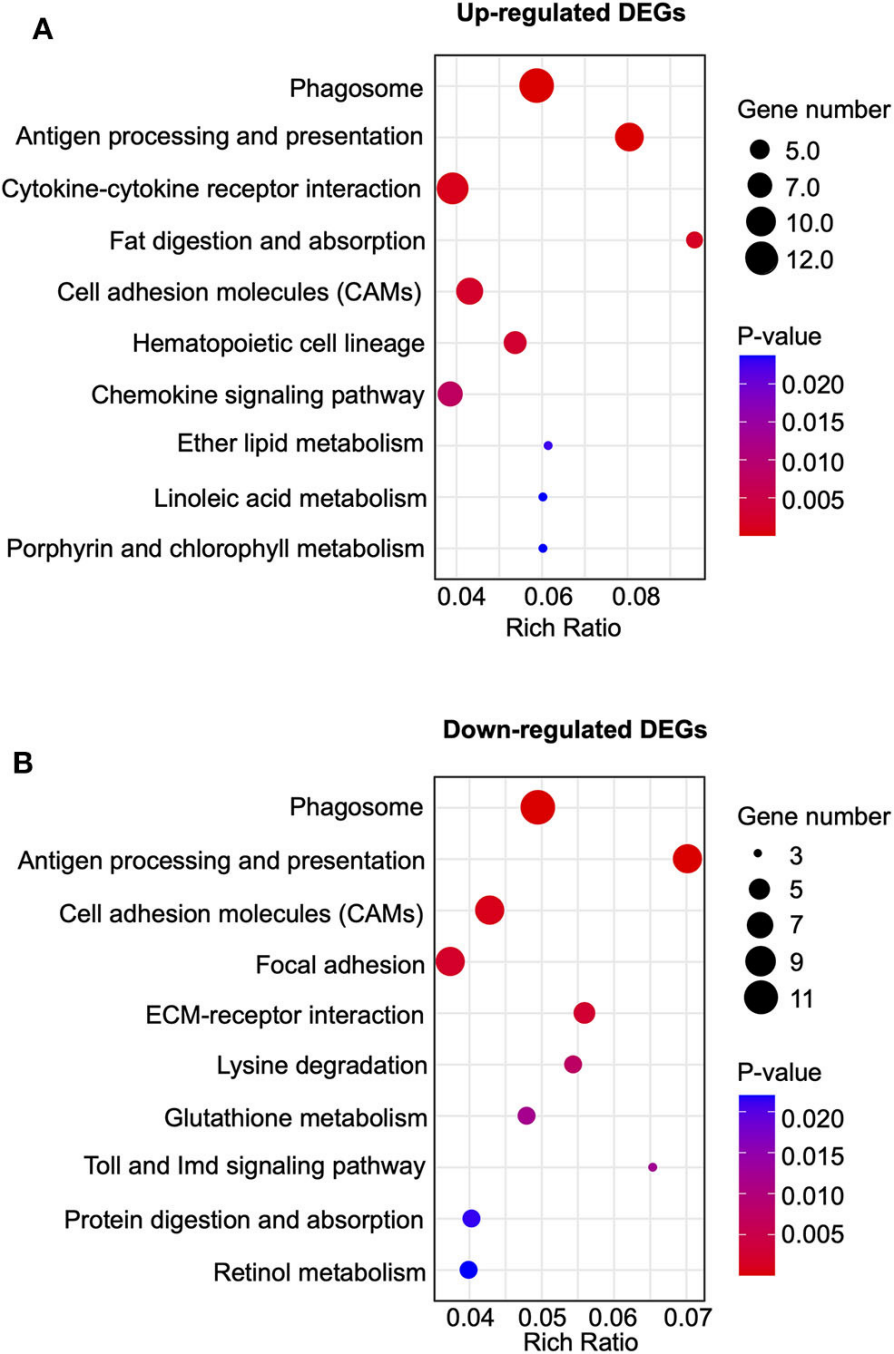
Kyoto Encyclopedia of Genes and Genomes (KEGG) pathway analysis of DEGs. **(A)** Bubble plots showing the top 10 significant pathways of upregulated DEGs. **(B)** Bubble plots showing the top 10 significant pathways of downregulated DEGs. Larger bubbles indicate higher number of genes. The color of each bubble reflects the significance (*p*-value).

Kyoto Encyclopedia of Genes and Genomes (KEGG) analysis was performed to further analyze the DEGs. KEGG analysis indicated that the up-regulated DEGs in CPIP+EA vs. CPIP group were exclusively involved in phagosome, antigen processing and presentation and cytokine-cytokine receptor interaction, etc. (Figure 6A). In addition, the down-regulated DEGs were exclusively involved in phagosome, antigen processing and presentation, cell adhesion molecules (CAMs), etc. (Figure 6B).

### RNA-Seq Data Validation via qPCR

**Figure 7:**
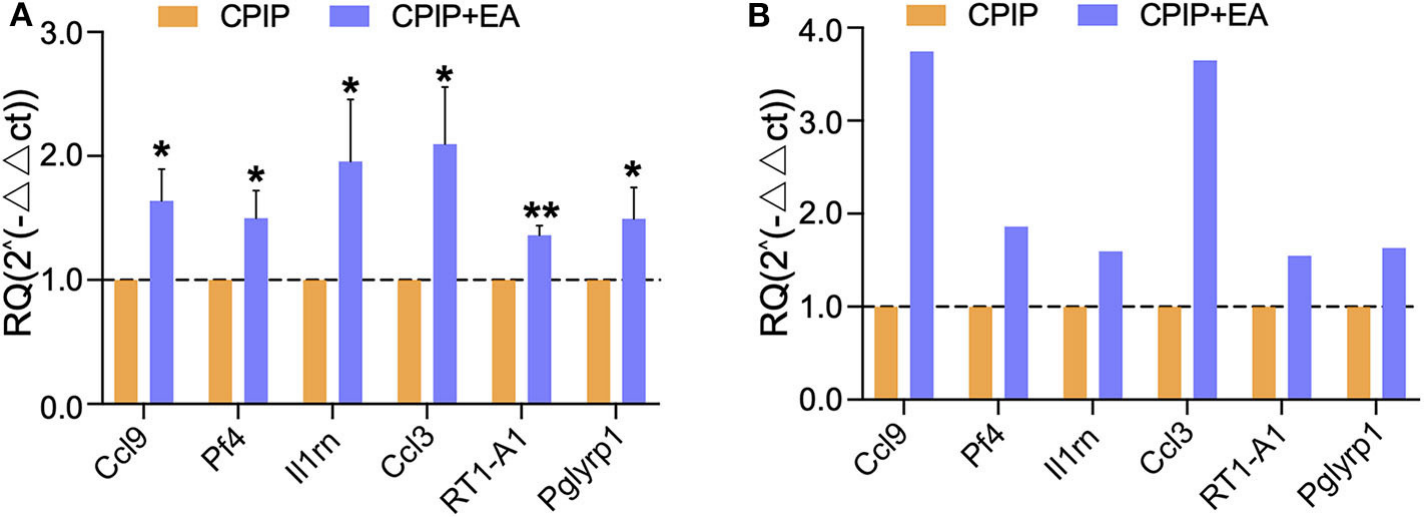
The validation of RNA-Seq results using qPCR. **(A)** The expression of representative genes in immune response detected by qPCR. **(B)** The expression of representative genes by RNA-Seq. *n* = 7 rat/group. *p < 0.05 and **p < 0.01 vs. with CPIP group. Student's *t*-test was used for statistical analysis.

Next, we set to examine the reliability of our RNA-Seq data using qPCR. We evaluated the up-regulated DEGs which were implicated in immune response identified by GO analysis. We found that the expression of 6 genes, including *Ccl9* [chemokine (C-C motif) ligand 9], *Pf4* (platelet factor 4), *Il1rn* (interleukin 1 receptor antagonist), *Ccl3* (C-C motif chemokine ligand 3), *RT1-A1* (RT1 class Ia, locus A1), and *Pglyrp1* (peptidoglycan recognition protein 1), were significantly up-regulated by EA treatment (Figure 7A). The trend was consistent with the results derived from RNA-Seq as shown in Figure 7B. Thus, the results of qPCR provide evidence showing that the RNA-Seq data for gene expression profiling was reliable.

### PPI Network Analysis of the DEGs Modified by EA Treatment

**Figure 8:**
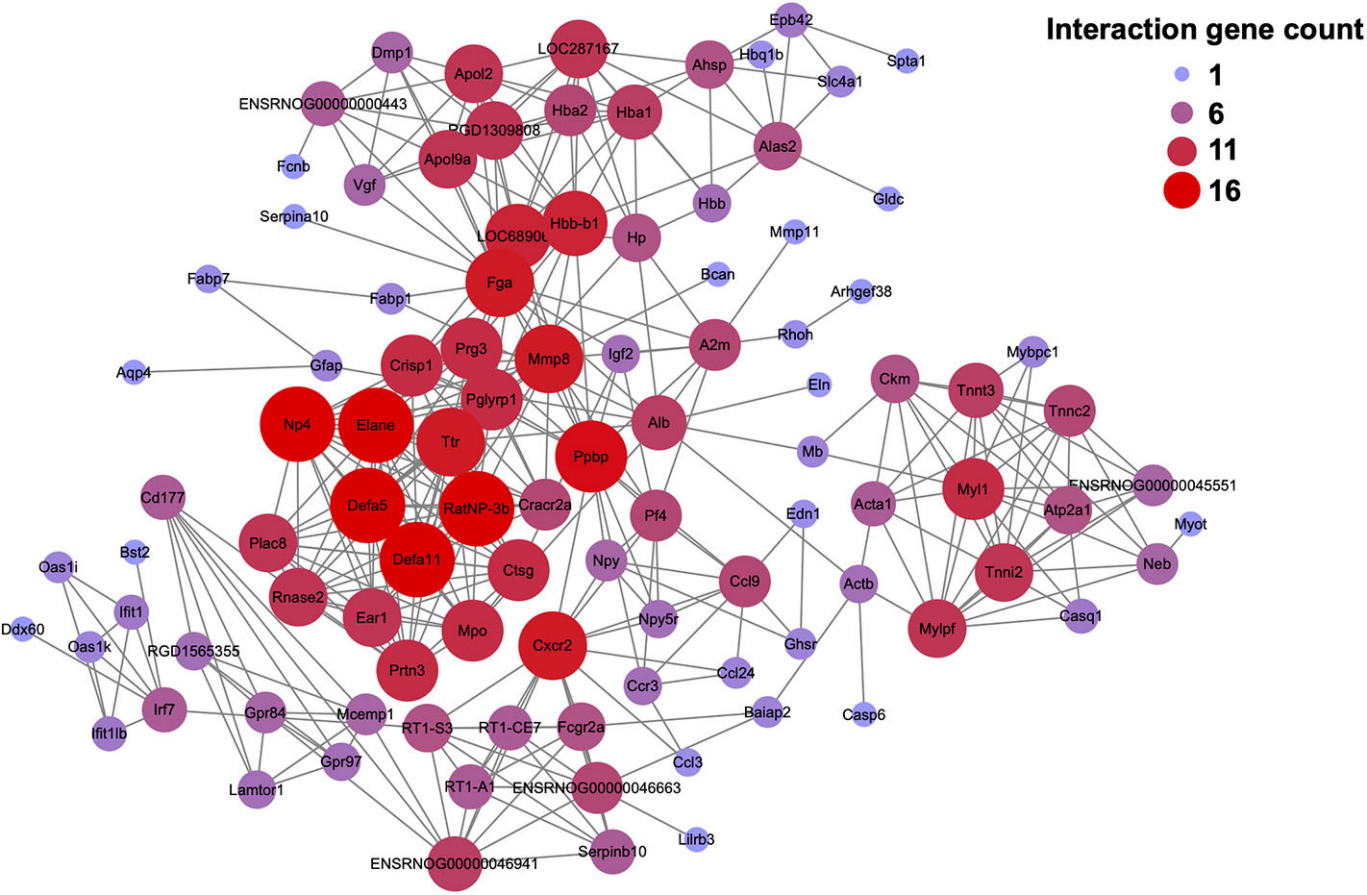
Protein-protein interaction (PPI) network analysis of the DEGs identified from CPIP+EA group vs. CPIP group. PPI network analysis of the DEGs increased or decreased in CPIP+EA group vs. CPIP group. Larger and red color circle reflects more interactions, whereas smaller and purple color circle means fewer interactions.

We further performed protein-protein interaction (PPI) network analysis of the DEGs modified by EA treatment (including 294 up-regulated and 258 down-regulated DEGs as shown in Figures 4A,B). The major hub genes derived from the PPI network analysis included: *RatNP-3b* (defensin RatNP-3 precursor), *Defa11* (defensin, alpha, 11), *Defa5* (defensin alpha 5), *Elane* (elastase, neutrophil expressed), *Ppbp* (pro-platelet basic protein), *Np4* (defensin NP-4 precursor), *Cxcr2* (C-X-C motif chemokine receptor 2), and *Mmp8* (matrix metallopeptidase 8), etc. (Figure 8).

## Discussion

In this work, we studied the anti-allodynic effects of EA on pain-related behaviors of the rat CPIP model. We performed RNA-Seq to study the gene expression profiles of ipsilateral L4-L6 DRGs innervating the hind limbs of CPIP model rats with and without EA treatment. We successfully identified a number of DEGs that are up- or down-regulated by EA treatment. We then studied the cellular and molecular functions of these identified DEGs via GO and KEGG enrichment analysis. Our results indicated that these DEGs were mostly involved in neutrophil chemotaxis, defense response to bacterium/fungus, immune response, and phagosome. We further validated the expression of some representative genes involved in neutrophil chemotaxis and immune response via q-PCR. Finally, PPI network analysis identified a bunch of hub genes that were modulated by EA treatment.

Mechanical allodynia is a prominent clinical feature observed in CRPS-I patients (32). The CPIP model rats developed obvious signs of mechanical allodynia, which well-recapitulates clinical observations in CRPS-I patients. Here, we observed that 100 Hz EA treatment applied right after model establishment significantly alleviated the mechanical allodynia of CPIP model rats. This result is consistent with our recent findings (15). Besides, our recent study also found that 100 Hz EA applied during the maintenance phase of CPIP model can also produce anti-allodynic effects (15). Therefore, these studies all suggest 100 Hz EA can serve as an effective and reliable method for treating CPIP model-related pain behaviors.

The mechanisms through which 100 Hz EA exerts remarkable relieving effect on CPIP-induced mechanical allodynia remain to be examined. In order to explore the genes and molecular pathways which may possibly participate in CPIP model-induced pain and those related to EA-induced pain relieving effect, we carried out RNA-Seq analysis of the ipsilateral DRGs that innervate the hind paw of EA's site of action. Our recent work identified that multiple genes showed expression changes in ipsilateral DRG neurons that innervate the hind limbs of CPIP model rats (26). Our present results are consistent with previous study, showing that CPIP model induced multiple gene expression changes in ipsilateral DRGs (26). We further found that EA treatment partially reversed the up- or down-regulated DEGs in CPIP model rats.

We then focused on the genes that are affected by EA treatment since these genes may be EA-responsive genes and implicated in EA's therapeutic effects. By means of GO analysis, we found that the top enriched biological process of up-regulated DEGs by EA treatment is neutrophil chemotaxis. Neutrophils are usually the first cell type to infiltrate into the tissues when there is noxious stimuli insult (33). Once infiltrated into tissues, they can initiate inflammation, oxidative burst, and clearance of pathogens via recruiting more macrophages (33–35). However, evidence also suggests that infiltrated neutrophils are also capable of driving resolution phase of inflammation via initiation of pro-resolving mechanisms, such as the release of pro-resolving lipid mediators and cytokine scavengers (36–38). These mechanisms can buffer the actions of pro-inflammatory cytokines and exert pro-resolving effects, which enable the conclusion of inflammatory responses (39). Therefore, the exact role of neutrophils in mediating the therapeutic effects of EA on CPIP model rats necessitates further study.

Another mostly enriched biological process exerted by EA we deduced from GO analysis is immune response. Our previous study found that immune response was the mostly enriched biological process in DRGs of CPIP model rats (26). Interestingly, repetitive treatment with EA provoked a further increase in the expression of immune response-related genes in DRGs of CPIP model rats (e.g., *Ccl24, Ppbp, Prg2, Ccl3, Ccl9, Cxcr2, Il1rn*, and *Ccr3*, etc.). This finding is consistent with two recent studies exploring the effects of spinal cord stimulation (SCS) on gene expressions in spinal cord of chronic pain model rats (40, 41). SCS is a therapeutic method for attenuating chronic pain (42). Repetitive SCS produced robust analgesic effect on chronic constriction injury (CCI) and paclitaxel-induced chronic pain model rats in these two studies. Further exploration of gene expression changes by RNA-Seq indicated repetitive SCS evoke an obvious increase of expression of many immune response–related genes in the spinal cord of these pain model rats (40, 41). It is usually considered that immune responses in DRGs participate in the initiation and maintenance phase of chronic pain (43). However, more and more evidence suggests that the immune responses can also serve to protect the injured area from further insult by engulfing pathogens, removing cell debris, exerting repair, and neuroprotection mechanisms (44–46). Therefore, EA and SCS may engage similar immune stimulatory effects to exert therapeutic effects on chronic pain conditions. The exact implications of up-regulated expression of immune response-related genes in DRGs after EA treatment of CPIP model rats needs further investigation.

CPIP model rats developed obvious mechanical allodynia in both ipsilateral and contralateral hind limbs, a phenomenon consistent with human (32) CRPS-I patients that exhibit bilateral hypersensitivity to mechanical stimuli (32). This phenomenon, in which trauma or inflammation on ipsilateral side elicits pain on contralateral non-injured side is called mirror-image pain (MIP). At the present, the detailed mechanisms underlying MIP remain largely unknown. It is believed that damage to peripheral or spinal tissues may produce a variety of inflammatory mediators and reactive oxygen species products and initiates inflammatory responses (47, 48). These products may be transported through body or cerebrospinal fluids to the contralateral site, affecting spinal cord, DRGs or peripheral nerves, and producing MIP (49). In addition, glial cell sensitization and their cross-talk in spinal cord are proposed to be involved in MIP (32, 47, 50). Our recent study, together with others, observed significant glial cell activation in bilateral sides of spinal cord dorsal horn of CPIP model rats (13, 18), suggesting glial cell activation may be involved in MIP of CPIP model rats. In the present study, our major focus is to explore the gene changes profile in ipsilateral DRGs after EA treatment. Therefore, we did not examine gene changes in contralateral DRGs of CPIP model rats and examined EA's effects. But these studies will be helpful for further elucidating the mystery underlying MIP of CRPS-I and unraveling EA's therapeutic mechanisms.

In summary, we found that repetitive EA treatment significantly attenuated the mechanical allodynia of the rat CPIP model, an animal model mimicking human CRPS-I. We further identified the genes and gene networks that were affected by EA treatment in DRGs of CPIP model rats. These results suggest that EA may alleviate pain response in CPIP model rats via regulating multiple genes in DRGs. Thus, our work may help to further advance the understandings of the molecular mechanisms underlying EA's therapeutic effects on CRPS-I-related pain.

## Data Availability Statement

The original contributions presented in the study are publicly available. The RNA-Seq dataset has been deposited into the National Center for Biotechnology Information's Gene Expression Omnibus repository with accession number GSE158560. This data can be found here: https://www.ncbi.nlm.nih.gov/geo/query/acc.cgi?acc=GSE158560.

## Ethics Statement

This animal study was reviewed and approved by the Animal Ethics Committee of Zhejiang Chinese Medical University (permission number ZSLL-2017-183).

## Author Contributions

JW, XZ, RC, XL, YL, HN, and DZ performed the experiments and analyzed the data. BoyuL and CY performed bioinformatics analysis of RNA-Seq dataset. XH and YJ coordinated with EA treatment. JF and BoyiL designed and supervised the study. BoyiL wrote the manuscript. All authors reviewed the manuscript.

## Conflict of Interest

The authors declare that the research was conducted in the absence of any commercial or financial relationships that could be construed as a potential conflict of interest.
